# Supplementary material for: Empirical methods for controlling false positives and estimating confidence in ChIP-Seq peaks
Source: BMC Bioinformatics. 2008 Dec 5;9:523. doi: 10.1186/1471-2105-9-523 (PMC2628906; doi:10.1186/1471-2105-9-523)
Supplement: Additional File 1 — A variety of html documents from the USeq web site detailing the available applications, their best usage, output file type descriptions, command line menus, etc. [file 1471-2105-9-523-S1.zip › USeqUserGuides/JavaDoc.zip!/JavaDoc/trans/misc/BestWindowScoreExtractor.html]

BestWindowScoreExtractor


|  |  |  |
| --- | --- | --- |
| **Overview** \| **Package** \| **Class** \| **Use** \| **Tree** \| **Deprecated** \| **Index** \| **Help** | |  |
| **PREV CLASS**   **NEXT CLASS** | **FRAMES**    **NO FRAMES**     **All Classes** |  |
| SUMMARY: NESTED \| FIELD \| CONSTR \| METHOD | DETAIL: FIELD \| CONSTR \| METHOD |  |


---


## trans.misc Class BestWindowScoreExtractor

```
java.lang.Object
  trans.misc.BestWindowScoreExtractor
```

---

``` public class BestWindowScoreExtractor extends java.lang.Object ```

Finds the best overlapping Window for each region and prints that score with the region information.
Built to associate a qPCR test result with the most overlapping window.

---

**Constructor Summary**

`BestWindowScoreExtractor(java.lang.String[] args)`
  
          For each validation region, finds the single test window that 'best' overlaps.


| **Method Summary** | |
| --- | --- |
| `static void` | `main(java.lang.String[] args)` |
| `trans.misc.BestWindowScoreExtractor.QPCRWindow[]` | `makeQPCRWindows(Window[] ws)` |
| `static Window[]` | `makeWindows(java.io.File txtFile)`             Converts a tab delimited txt file of chr, start, stop, scores (multiple permitted or none) into an array of Windows. |
| `static void` | `printDocs()` |
| `void` | `processArgs(java.lang.String[] args)`             This method will process each argument and assign any new varibles |
| `static double` | `windowMidPoint(Window w)` |

**Methods inherited from class java.lang.Object**

`equals, getClass, hashCode, notify, notifyAll, toString, wait, wait, wait`

**Constructor Detail**

### BestWindowScoreExtractor

```
public BestWindowScoreExtractor(java.lang.String[] args)
```

:   For each validation region, finds the single test window that 'best' overlaps.


**Method Detail**

### windowMidPoint

```
public static double windowMidPoint(Window w)
```

---


### processArgs

```
public void processArgs(java.lang.String[] args)
```

:   This method will process each argument and assign any new varibles

---


### printDocs

```
public static void printDocs()
```

---


### main

```
public static void main(java.lang.String[] args)
```

---


### makeQPCRWindows

```
public trans.misc.BestWindowScoreExtractor.QPCRWindow[] makeQPCRWindows(Window[] ws)
```

---


### makeWindows

```
public static Window[] makeWindows(java.io.File txtFile)
```

:   Converts a tab delimited txt file of chr, start, stop, scores (multiple permitted or none)
    into an array of Windows.


---


|  |  |  |
| --- | --- | --- |
| **Overview** \| **Package** \| **Class** \| **Use** \| **Tree** \| **Deprecated** \| **Index** \| **Help** | |  |
| **PREV CLASS**   **NEXT CLASS** | **FRAMES**    **NO FRAMES**     **All Classes** |  |
| SUMMARY: NESTED \| FIELD \| CONSTR \| METHOD | DETAIL: FIELD \| CONSTR \| METHOD |  |


---
